# Is It Time to Retire Aldosterone Suppression Testing?

**DOI:** 10.1093/ajh/hpaf163

**Published:** 2025-08-28

**Authors:** Gregory A Kline, Alexander A Leung, Dennis Orton, James MacFarlane, Mark Gurnell

**Affiliations:** Department of Medicine, Cumming School of Medicine, University of Calgary, Calgary, Alberta, Canada; Department of Medicine, Cumming School of Medicine, University of Calgary, Calgary, Alberta, Canada; Department of Pathology and Laboratory Medicine, BC Children’s and Women’s Hospital, Vancouver, British Columbia, Canada; Department of Medicine, University of Cambridge & Cambridge University Hospitals NHS Foundation Trust, Cambridge, UK; Department of Medicine, University of Cambridge & Cambridge University Hospitals NHS Foundation Trust, Cambridge, UK

**Keywords:** aldosterone, blood pressure, hypertension, primary aldosteronism, secondary hypertension

## Abstract

Primary aldosteronism (PA) is the most common endocrine hypertension. For decades, PA diagnosis has required proving nonsuppressibility of aldosterone following maneuvers modulating the renin-angiotensin-aldosterone pathway. This includes oral salt suppression, intravenous saline suppression, captopril suppression, and others. Grounded in rational first principles from pathophysiologic considerations and small, early pathophysiologic studies following Conn’s initial PA description, such testing has been widely recommended. However, a modern understanding of PA pathophysiology and critical appraisal of diagnostic test studies suggest that traditional suppression testing is not suited to diagnosis or disease definition. There are four main problems recently raised regarding aldosterone suppression testing: (i) PA is now known to exist along a continuous biochemical spectrum and it is scientifically impossible to draw a single, diagnostic threshold within this continuum. (ii) Aldosterone assay uncertainty is sufficiently large to yield contradictory final diagnoses when applied to a threshold during suppression testing. (iii) The pathophysiology of PA is multifactorial with multiple mechanisms not necessarily relevant to salt and volume loading tests. (iv) Finally, meta-analysis of suppression testing studies demonstrated extensive biases and confounders, which have overestimated the diagnostic value. A recent prospective, blinded study of saline suppression for PA diagnosis defined by medical or surgical response to PA-targeted therapy showed no discrimination according to nadir aldosterone level. Given the clinical value of a PA diagnosis and the high prevalence of the disease, modern evidence suggests that aldosterone suppression testing should now be retired from the diagnostic pathway; new ways of approaching the definition of PA are provided to spur further discussion.

## Graphical Abstract

**Graphical Abstract:**
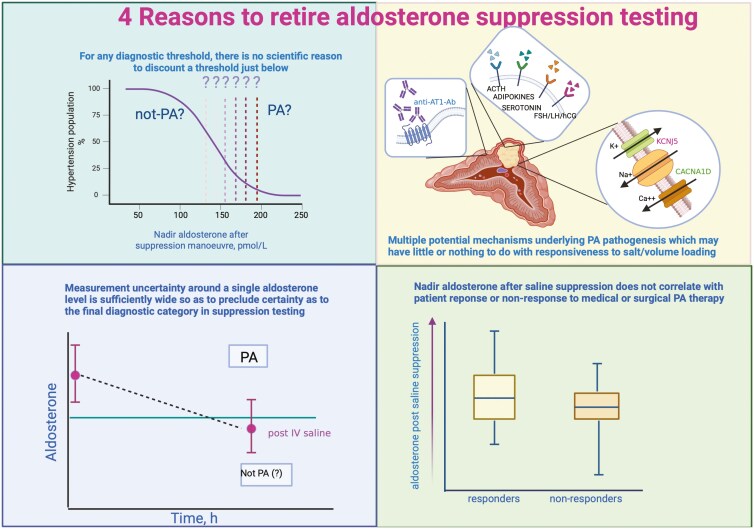
Licence: Created in BioRender. Kline, G (2025). https://BioRender.com/4cwv6zg

Primary aldosteronism (PA) is now widely recognized as the most common form of endocrinopathy causing chronic hypertension^1^ with a plethora of poor health outcomes directly arising from both the hypertension and aldosterone excess.^2,3,4^ Even with the most conservative estimates of disease prevalence, it is likely that there are millions of people worldwide who have PA but are unaware, undiagnosed, and untreated.^5,6^ For example, in one highly integrated provincial health system with universal health insurance coverage and near complete capture of all health encounters, laboratory tests, diagnostic imaging, and medication dispensation records, it was shown that less than 1% of hypertensive individuals were ever screened for PA. Among the few who were screened, possible PA was discovered in more than 20% and yet few cases were ever offered any additional diagnostic testing; fewer still any disease-specific therapy.^7^ A recent “direct-to-patient” free, online PA screening campaign intended to remove barriers to PA screening in the United States, showed that even after case detection through this method, downstream follow-up or treatment events were very uncommon.^8^ This demonstrates that in addition to low initial screening rates, clinical inertia or uncertainty is a significant barrier to most patients receiving the care needed to improve their long-term outcomes.^9^ One probable barrier is the current belief that some form of confirmatory testing, in the form of dynamic attempts to suppress aldosterone, is required before a diagnosis of PA can be assigned and treatment considered, but this requisite is not well-substantiated. The concept of “confirmatory testing” in medicine is well-established and usually important for many diseases; an example would be screening mammography and then core needle biopsy for suspicious lesions. However, the accuracy, reproducibility, and predictive value of any confirmatory test must be very high if it is to reliably confirm a diagnosis, otherwise it may simply add confusion to an uncertain clinical picture. The time has come to reexamine this paradigm in PA, to determine whether it is still needed for PA diagnosis and whether its use ultimately aids or hinders the average patient’s access to diagnosis and therapy.

## THE SPECTRUM OF ALDOSTERONE CONFIRMATORY TESTS

Over time, numerous so-called “confirmatory tests” for PA have been proposed, studied, and popularized. In their simplest form, this could mean the measurement of plasma aldosterone following an oral dose (25–50 mg) of captopril^10,11^ or the measurement of urinary aldosterone concentration following 72-h of oral salt loading.^12,13^ More complex testing involves aldosterone measurement following several hours of 0.9% intravenous saline infusion,^14,15^ measurement of renin following intravenous furosemide loading^16^ or even inpatient admission for several days of monitored fludrocortisone administration (with concomitant potassium therapy).^17^ The simplest confirmatory tests may be feasible in a nonexpert outpatient setting, provided that the lab is able to offer sequential blood draws but even then, the administration of medications (slow sodium tablets, 0.9% sodium chloride solution or captopril) provides an additional regulatory and logistic hurdle. Complex testing requires a dedicated unit with specialized staff and intravenous access, if not formal admission to hospital with inpatient monitoring. It is therefore unsurprising that diagnostic drop-off happens early on in the recommended processes.

## WHERE DID ALDOSTERONE SUPPRESSION TESTING COME FROM?

Students of the history of PA will recognize that the index case of “Conn syndrome”^18^ and many of the early cases published thereafter^19^ did not incorporate the notion of either screening or confirmatory testing. Initial cases were recognized clinically, likely due to their florid nature (e.g., very severe hypertension, hypokalemic muscle paralysis, urinary concentrating defect, proteinuria), and supporting measurements of aldosterone. Possibly reflecting reporting bias, many subsequent published cases looked similar, defining the syndrome by its most extreme manifestations. Nonetheless, with characteristic insight, Conn predicted that PA would eventually prove to be common and that cases would emerge in which the phenotype would be a eukalemic version indistinguishable from what might be called essential hypertension at the bedside.^20^

A dichotomous paradigm of disease emerged, based on the presence or absence of a florid phenotype. Small groups of patients, who it was claimed had overt PA, underwent physiologic studies, including oral and intravenous salt loading,^21^ clinical response to spironolactone,^22^ oral potassium loading,^23^ and positional changes and fludrocortisone loading.^24,25^ Not surprisingly, it was straightforward to show measurable pathophysiologic differences between these cases and uncomplicated controls. These reports demonstrated that such testing “confirmed” the anticipated diagnosis among those who clearly had it. Conn’s own helpful addition was to stress that PA, at the least, must be characterized by low renin, in the face of an abnormally elevated aldosterone.^26^ The remaining question was whether this simplistic view would be sufficient to justify an intervention like adrenalectomy. The classical paradigm of PA has therefore evolved since, upon the following four related assumptions:

1. PA is something unique and different than other causes of hypertension.
2. A hypertensive person may or may not have PA (a binary state).
3. All screening tests, by nature, can have false positives and false negatives.
4. Additional biochemical testing is needed to secure a PA diagnosis after a positive screening test.

A major controversy in PA is therefore, whether confirmatory testing would be adopted today, based on the existing evidence, had it not evolved at a time where only florid cases were recognized. If not, then the second question must be whether it is time to retire the old diagnostic paradigm in favor of something that is better suited to the modern understanding of this condition. In this review, we shall examine several problems that have arisen for the continued classical understanding of PA and the role of confirmatory testing.

## PROBLEM #1: ALDOSTERONE-MEDIATED HYPERTENSION EXISTS ON A CONTINUUM

**Figure 1.**
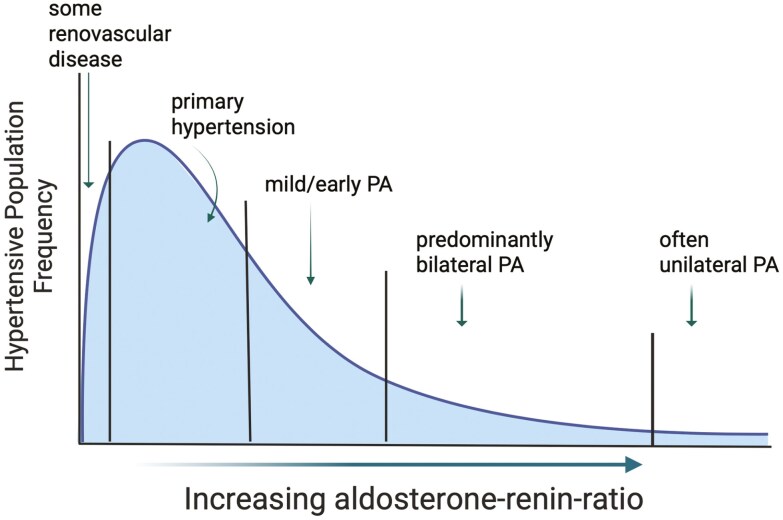
The spectrum of renin-aldosterone dysregulation in chronic hypertension. Conceptual schematic demonstrating the spectrum of aldosterone and renin relationships and various recognized forms of hypertension. Very high renin hypertension with low aldosterone-renin-ratio may be seen in some renovascular disease and most cases of primary hypertension. Increasing dissociation of aldosterone from renin, particularly with progressively lower renin measures (high aldosterone-renin-ratio) suggests increasing aldosterone autonomy. The most severe forms of aldosterone autonomy in hypertension may represent a small fraction of cases with solitary aldosterone-producing adenomas. Created in BioRender. Kline, G. (2025) https://BioRender.com/hbtet6w

The presence of sustained, chronic hypertension is not merely an epidemiologic risk factor for cardiovascular disease in large populations; it represents the presence of pathology affecting the renin-angiotensin-aldosterone system (RAAS) within the individual. Although mechanistically complex with both genetic predisposition and environmental factors contributing, chronic hypertension ultimately represents *a response to* disordered renal sodium handling.^27^ This is broadly associated with RAAS hyperactivity which may be simplistically explained in terms of inappropriate renin-angiotensin II activity (leading to peripheral vasoconstriction) or inappropriate mineralocorticoid activity (resulting in sodium retention) or both.^28,29^ At the extremes of the spectrum are those with very high inappropriate renin production, as in renovascular narrowing or obstruction^30^ vs. those with high, inappropriate primary aldosterone excess, collectively called PA (**Figure 1**).

Represented schematically as a continuum of renin or aldosterone-driven pathophysiology, phenotypes within both tails may be further considered as being mild, moderate, or severe. In the mid-part of the continuum, it may be difficult to pinpoint any single dominant endocrine pathway—which does not exclude some degree of aldosterone autonomy. There is epidemiologic evidence in support of this model, including the Framingham Offspring study where researchers were unable to find any evidence of a bi-modal distribution in aldosterone-to-renin ratios in hypertensive patients, but rather a continuum.^31^ Vaidya *et al*. have demonstrated a clear continuum of ARR results even among patients with unilateral PA, including ARR levels below traditional screening thresholds.^32^ Other studies of inappropriate aldosterone excess have demonstrated a clear association between renin-independent aldosterone levels and progressively severe hypertension (representing the aldosterone tail of the distribution). Mild aldosterone excess was still present in some patients with prehypertension or mild hypertension, a group traditionally assumed to have “essential hypertension” unrelated to mineralocorticoid dynamics.^33,34^

The existence of aldosterone excess across a biochemical and clinical continuum invites application of the philosophical Sorites paradox: it is impossible to reliably and scientifically define any incontrovertible threshold along a continuum that can truly discriminate between the presence and absence of disease.^35^ If PA is defined as a “post-suppression” aldosterone level of 170 pmol/L, then why not 169 pmol/L and if 169 pmol/L then why not 168 pmol/L and so on. Thus, no matter where one draws the defining line for PA, the conclusion is inherently arbitrary, which is problematic for a test that is supposed to confirm or exclude a categorization.

## PROBLEM #2: THE PHYSIOLOGY OF SUPPRESSION TESTS NO LONGER FITS OUR UNDERSTANDING OF HOW PA ARISES

At its historical core, confirmatory testing is conceptually aimed at demonstrating aldosterone autonomy, independent of renin regulation. Thus, confirmatory tests to date have focused upon dynamic modulation of classical RAAS physiology and the relationship of sodium, renin, and angiotensin II to aldosterone secretion. For example, the posture test and the furosemide test focus upon whether renin may become nonsuppressed. The fludrocortisone and oral salt loading test are based upon ability to show aldosterone persistence despite maximal sodium/mineralocorticoid suppression of renin. The captopril suppression test depends upon a persistence in aldosterone levels upon blocking the effect of renin-AII signaling. The intravenous saline test is predicated upon suppression of aldosterone (and renin) via intravascular volume expansion and sodium loading.

However, much research in PA has now demonstrated that renin-independent aldosterone secretion may also be a function of ACTH,^36^ serum potassium levels,^37^ ectopic/aberrant adrenal hormone receptor ligands,^38^ stimulatory anti-AII antibodies,^39^ somatic and germline mutations in calcium channels or Na/K ATPases,^40^ adipokines,^41^ steroidogenic and steroid metabolism enzyme polymorphisms^42^ and functional variants of the mineralocorticoid receptor or downstream ENaC signaling.^43^ We have previously demonstrated in a multisite study using both immunoassay and LCMS/MS methods, a substantial proportion of PA patients, including those with confirmed unilateral disease, have aldosterone levels which can become highly “suppressed” by intravenous sedation alone—a maneuver which would appear to be wholly independent of the classic mechanisms of RAAS suppression or blocking.^44^ In a similar fashion, Parsook *et al*. recently demonstrated that the intravenous saline suppression test induces significant hypocalcemia in all subjects, and the decrease in calcium is proportional to the decrease in aldosterone and inversely proportional to the predicted body-size-related volume of distribution of the saline with related increase in acute renal calcium clearance. They concluded that the apparent aldosterone suppression may have more to do with the induction of hypocalcemia (and effect upon calcium channel-mediated aldosterone secretion).^45^ If this ends up being a function of saline volume-to-body-size, then it suggests that our traditional understanding about what saline infusion was doing to aldosterone levels may be an oversimplification. Therefore, the relationship between the observed aldosterone response to saline loading and underlying PA pathophysiology remains uncertain. Finally, Markou *et al*. have demonstrated in some individuals with hypertension, a hyper-responsiveness to minute doses of ACTH and exercise, yielding high aldosterone and low renin.^36^ Although it broadly fits the pattern of renin-independent aldosterone secretion (and such patients were MRA-sensitive), it is unclear how such cases might respond to suppression tests that are unrelated to their ACTH-dependent mechanism of renin-independent aldosterone secretion.

## PROBLEM #3: MEASUREMENT UNCERTAINTY UNDERMINES STRICT THRESHOLD DEFINITIONS OF ALDOSTERONE EXCESS

In the absence of florid hypokalemic forms, cases of PA are defined by laboratory measurements of aldosterone and renin. It is well-established that all such measures are prone to a certain degree of measurement uncertainty related to both biological variation (in health and disease) as well as method-specific uncertainty.^46^ Yang and colleagues have demonstrated the practical consequences of measurement uncertainty when applied to the aldosterone-renin ratio. In their population of 162 patients with PA where two aldosterone-to-renin-ratio (ARR) measures were performed, 38% had at least one result below the threshold used to define a positive screening result, indicating that a single test of ARR may be diagnostically misleading in a proportion of patients ultimately found to have PA.^47^ This same principle must also be applied to aldosterone suppression testing.

For example, if a lab reports a mean analytical imprecision (CVa) of 9.5% (as would be approximately the case in many labs) for its aldosterone immunoassay, then the 95% CI around any single aldosterone measure would be 1.96*CVa = result ± 19%. Thus, for an interpretive aldosterone threshold of 140 pmol/L, even results as low as 113 pmol/L must be considered potentially compatible with nonsuppression. This aldosterone level is only slightly above the lowest reporting limit for some laboratory’s aldosterone measures; effectively diagnosing PA in every case where the aldosterone does not fall below the lowest reporting limit. In turn, this would make almost every confirmatory test “positive” in which case, it adds little to the original screening result.

## PROBLEM #4: THE CLINICAL EVIDENCE BASE FOR SUPPRESSION TESTING HAS QUESTIONABLE VALIDITY

Whatever its theoretical or practical constraints, the final arbiter of test usefulness will be its performance in real-world scenarios. This is why the existing confirmatory tests have been subjected to multiple attempts at validation. Several summaries and cross-comparisons have attempted to synthesize conclusions from published studies^48,49^ but an important prior step should be to rigorously analyze the various study designs that make up the existing literature; problematic study designs are more likely to yield results of uncertain validity. A 2022 systematic review and meta-analysis of confirmatory test literature to date included formal diagnostic evidence assessment tools to help answer this question. It was concluded that from 55 included studies, nearly half used a single-gate design (i.e., compared patients with PA to those who were never suspected to have PA, such as healthy volunteers), nearly 25% used the experimental test [the confirmatory test] as its own reference standard, and three-quarters failed to apply any uniform case verification across all subjects. On meta-regression analysis, the authors demonstrated that these faulty study designs would be predicted to overestimate the diagnostic accuracy of the study test in question by as much as 7-fold.^50^ And, applied to a model population reflecting expected PA prevalence in a tertiary hypertension clinic, the number of missed PA cases (false negatives) exceeded the number of over-diagnosed (false positive) cases for all test protocols. Perhaps not surprisingly, Monticone and colleagues showed that a substantial number of surgically curable unilateral PA cases appear over time among patients with initial negative confirmatory testing.^51^ This may be due to disease progression or may reflect the overall inaccuracy of the initial confirmatory test—but either way, demonstrates that a “negative” confirmatory test does not reliably exclude PA.

Although meta-analysis is a useful tool to collect and condense the observations of prior studies, the results are still limited in validity when the primary sources have design problems. To overcome the inherent limitations of retrospective studies, we recently reported the results of the first prospective, blinded, outcome-based study examining the relationship between nadir serum aldosterone during saline suppression and patient response to aldosterone-targeted therapy (MRA or surgery).^52^ Patients with high pretest probability for PA (hypertension with high ARR) underwent seated intravenous saline suppression testing but the clinicians were blinded to the results. All patients went on to adrenal vein sampling and either surgical or medical therapy and final outcomes were adjudicated by the international PASO criteria (surgical)^53^ or similar to the recent PAMO criteria (medical).^54^ The results demonstrated that there was no nadir aldosterone threshold, irrespective of assay (immunoassay or LCMS/MS) which reliably predicted response to aldosterone-targeted therapy of any kind. Many patients with IVSS-aldosterone levels below commonly recommended thresholds still had unilateral adrenal disease and robust responses to PA-directed therapy. These results confirmed the conclusions of the prior meta-analysis; reports claiming to show diagnostic discrimination from so-called confirmatory tests were likely explained by biased, retrospective study design. In a blinded, prospective study design the power of a common confirmatory test could not be replicated and if anything, results showed that continued use of this nonpredictive test might actually cause harm by falsely pointing physicians away from an intervention that could have been highly impactful.

In summary, if various confirmatory test protocols had not been “baked” into the evolving initial understanding of PA, it is unlikely that such tests would be adopted today on the basis of the existing evidence. Whilst the challenges associated with confirmatory testing have been outlined, we need to acknowledge potential difficulties for the diagnosis of PA in their absence.

## QUESTION 1: HOW WILL WE DEAL WITH THE POSSIBILITY OF A FALSE POSITIVE ARR?

This question pertains to the first situation that led to the birth of the confirmatory testing paradigm. The often-over-looked corollary is that the ARR is an endocrine test like any other, and we are accustomed to thinking about false positives and false negatives for all such tests. This way of thinking arises from conceptualizing a test in a 2 × 2 table format where false negatives, true negatives, false positives, and true positives are collected and categorized with respect to an agreed reference standard. In PA, as outlined above in Problem 1, this might not be possible, realizing that PA is a spectrum of inappropriate mineralocorticoid excess. Therefore, one cannot speak scientifically about true positives and false positives in ARR or confirmatory tests; one may only speak about confounding in preanalytical conditions, assay problems, and postanalytical misinterpretations. Attention to preanalytical conditions (including intra-individual variability^55^) continues to be important, as are assay specifics (such as immunoassay vs. LCMS/MS). However, we propose that the ARR should be considered an observation (akin to a symptom or sign of disease), rather than a dichotomous test in the traditional sense. The difference is that an observation, if valid, requires clinical context and judgment for interpretation. By itself, it does not necessarily diagnose or exclude disease—that is the responsibility of the clinician who has seen the patient. Therefore, when the question pertains to the presence of “PA” following a laboratory test, there are no false positives or false negatives, just an appreciation of the aldosterone level, the renin level, and degree of separation between the two. A very high ARR in a hypertensive person, collected under valid conditions, strongly points to renin-independent aldosterone autonomy; a low ARR, on the other hand, points away from this mechanism—but the final interpretation requires proper clinical context or repeat measurement.

## QUESTION 2: WHAT WOULD CONTEXT-INFORMED PA DIAGNOSIS LOOK LIKE?

In the emerging paradigm of PA, the “entry point” of measuring an aldosterone-renin-ratio depends upon the perception that such measurement may help address a clinical problem, whether resistant hypertension, hypokalemia, young-onset hypertension, etc. When clinician and patient agree that there is a problem to be solved, a valid measurement of the ARR may provide useful information to guide next steps. The key is always the full context of the patient. A patient with unprovoked hypokalemia and hypertension will always have a very high probability of fitting a label of PA, even if the ARR is not markedly high. A patient with very elevated ARR will have a high probability of fitting a label of PA even if they lack hypokalemia or resistant hypertension. A patient with repeatedly low ARR will have a low chance of fitting a label of PA unless the clinician can identify a reason to question the validity of the measurement.

## QUESTION 3: TO WHAT EXTENT DOES A FORMAL LABEL OF PA AID IN CLINICAL PROBLEM-SOLVING?

In years past, a label of PA was the necessary entry point before treatment with a MRA would be considered. Without such a label, the default assumption was that hypertension should be treated according to “conventional” drug therapy (such as renin-angiotensin system blockers, thiazide diuretics, and calcium channel blockers) based on clinical practice guidelines. However, the high proportion of patients with resistant hypertension^56^ and strong randomized controlled trial evidence to show that spironolactone is highly effective in such patients^57^ has opened the door to the broad use of MRAs, even in the absence of a formal label of PA.^58^ A higher ARR result appears to predict MRA response^59,60^ and therefore the value of formally labeling a person who has difficult-to-control hypertension with “PA” seems small if it does not alter the choice of optimal drug therapy.

In the modern paradigm of PA, it is possible that decision-making around aldosterone and hypertension will be more focused upon whether and to what extent one embarks on a search for unilateral aldosterone excess that is potentially amenable to surgery. Thus far, this outcome also does not seem to be determined by the results in confirmatory testing^52^; biochemical prediction rules have been generated but external validation has been disappointing.^61,62^ Work continues on AVS patient selection models that help to define the best constellation of factors which correlate not just with lateralization but lateralization probability strata.^63^ This kind of pretest probability generator can then be tailored to health system resources and patient goals and expectations, rather than a rigid algorithm that is expected to apply globally.

## QUESTION 4: WHERE DOES CONFIRMATORY TESTING FIT IN THE NEW 2025 PA GUIDELINES?

The Endocrine Society published new PA guidelines this year and curiously have repositioned confirmatory testing (now officially called aldosterone suppression testing) less as a diagnostic standard for PA and more as a test that is purported to help select patients who should undergo adrenal vein sampling.^64^ This represents a philosophical change in the way historical confirmatory testing has been viewed. Broadly speaking, this positioning of the test might theoretically select PA patients for AVS by using suppression testing as a means to delineate different levels of PA severity. In turn, this assumes that AVS patient selection should be informed by such stratification and that suppression testing actually performs as intended in that light. Both assumptions have yet to be tested in prospective, blinded studies which is probably why the guideline recommendation is qualified as “very low certainty.”

## UNCERTAINTY 1: IF ORDERING THE ARR IS A CONTEXT-INFORMED DECISION, IS THERE ANY FUTURE ROLE FOR ARR SCREENING IN ALL PERSONS WITH HYPERTENSION?

While ARR screening has been recommended in some guidelines^64,65^ and surrogate measures of CV disease show a possible link to higher ARR measures,^34^ a prospective study using MRA as ARR-guided first-line therapy in all-comers with hypertension has yet to be done. If such a study is completed and shows usefulness to early MRA intervention, the paradigm of PA will need to be revised once more to consider PA biochemical screening as a necessary step to select the best possible initial treatment.

## UNCERTAINTY 2: WHAT IS THE ROLE OF RENIN-GUIDED MRA THERAPY IF PA BECOMES A LESS-SPECIFICALLY DEFINED ENTITY?

Similar to the question about screening, there is some early evidence to support renin-guided MRA therapy dosing in patients who are deemed to have PA as a surrogate of sufficient MR antagonism and volume contraction.^66^ Based on retrospective data, these observations necessarily pertain to historical paradigms of PA which in turn, likely represent the more severe end of the spectrum. Whether renin-guided therapy is advantageous to all hypertension patients with high ARR awaits prospective clinical trials and if so, may require a further shift in the way that all hypertension is understood. Until then, endocrinologists will have to decide on an individual patient basis how much their disease aligns with a historical, more severe version of PA and whether to apply renin-titrated MRA therapy.^67^

## UNCERTAINTY 3: HOW WILL RESEARCHERS DEFINE PA FOR THE PURPOSE OF COMPARING OR COLLABORATING ON PA STUDIES?

Harmonization of diagnostic standards in most diseases offers obvious benefits

Collaborative clinical trials may be conducted with disease definitions that are recognized and consistent; knowledge translation efforts are easier when research methods and case definitions are reproducible in real-world practice. In the present review, we are not arguing that biochemical definitions of PA should be abandoned altogether, but only that they should be reasonable and reproducible (i.e., two high measures of ARR in a proper context) and that researchers and reviewers should be prepared to recognize a certain degree of diagnostic flexibility between different centers. This is already very much the case for PA, with no global agreement on the ideal ARR, suppression test or AVS interpretation.^50,68,69,70,71^ Our proposed paradigm codifies the idea that minor variations in PA definition between different centers are quite acceptable, including case definitions that do not use any form of aldosterone suppression testing. Standards for PA treatment outcomes are more important in terms of comparing and assessing research, and these already exist. If a sensible PA case definition is used in a clinical trial with outcomes reported according to the consensus standard, then the study should be considered potentially valid.

Although a PA paradigm defined by very severe cases and confirmatory testing has likely served well for the past half-century, with increasing experience and scientific observation, it has become apparent that this older paradigm no longer fits the “inappropriate aldosterone-mediated” hypertension seen today. Reliance upon an older diagnostic paradigm is problematic for philosophical, aetiopathogenic, and measurement-related reasons.

The historical published evidence in support of confirmatory testing is likely insufficient to justify their continued use in practice, especially given the associated complexity and cost, which present major barriers to diagnosis for most patients.^6^ Whilst the high positive predictive value of confirmatory tests may be of benefit to clinicians in certain circumstances, the poor accessibility of the tests and the high rates of false negatives make them inappropriate for where they are currently positioned in guidelines/diagnostic algorithms.

We have highlighted a paradigmatic change in PA whereby the use of a single unique diagnostic algorithm is exchanged for a context-informed and shared decision-making process that interprets the ARR as an observation relevant to disease mechanism rather than a traditional dichotomous endocrine test. It is hoped that this newer understanding may offer more leeway to clinicians to consider the role of aldosterone excess in their hypertensive patients.
